# Fast entropy-based CABAC rate estimation for mode decision in HEVC

**DOI:** 10.1186/s40064-016-2377-0

**Published:** 2016-06-17

**Authors:** Wei-Gang Chen, Xun Wang

**Affiliations:** School of Computer and Information Engineering, Zhejiang Gongshang University, Hangzhou, 310018 China

**Keywords:** High efficiency video coding, Mode decision, Rate-distortion optimization, Context-adaptive binary arithmetic coding, Rate estimation

## Abstract

High efficiency video coding (HEVC) seeks the best code tree configuration, the best prediction unit division and the prediction mode, by evaluating the rate-distortion functional in a recursive way and using a “try all and select the best” strategy. Further, HEVC only supports context adaptive binary arithmetic coding (CABAC), which has the disadvantage of being highly sequential and having strong data dependencies, as the entropy coder. So, the development of a fast rate estimation algorithm for CABAC-based coding has a great practical significance for mode decision in HEVC. There are three elementary steps in CABAC encoding process: binarization, context modeling, and binary arithmetic coding. Typical approaches to fast CABAC rate estimation simplify or eliminate the last two steps, but leave the binarization step unchanged. To maximize the reduction of computational complexity, we propose a fast entropy-based CABAC rate estimator in this paper. It eliminates not only the modeling and the coding steps, but also the binarization step. Experimental results demonstrate that the proposed estimator is able to reduce the computational complexity of the mode decision in HEVC by 9–23 % with negligible PSNR loss and BD-rate increment, and therefore exhibits applicability to practical HEVC encoder implementation.

## Background

High efficiency video coding (HEVC), which is the newly developed video coding standard, follows the so-called block-based hybrid coding architecture (Sullivan et al. 2012). HEVC aims at providing higher coding efficiency and making the codec better parallelization than the prior standards. The reference software HM (https://hevc.hhi.fraunhofer.de/svn/svn-HEVCSoftware) has achieved the expected performance, but at the cost of some high computational coding tools, including quadtree based coding unit (CU), large and asymmetric prediction unit (PU), residual quadtree based transform unit (TU) (Ohm et al. 2012; Bossen et al. 2012; Kim et al. 2012; Corrêa et al. 2012; Pan et al. 2014).

Mode decision, which controls how a coding tree unit (CTU) is coded with CUs with variable block sizes and prediction modes, is an essential process in HEVC. To achieve the best performance, HEVC seeks the best coding tree configuration, the best PU division and the prediction mode, etc., by evaluating the rate-distortion (R-D) functional where a distortion term is weighted against a rate term using a “try all and select the best” strategy (Pan et al. 2014).

The rate term in the R-D functional represents an estimate for the number of coded bits produced by the entropy coder. Unlike H.264/AVC, the context adaptive variable length coding (CAVLC) is not supported in HEVC. It only defines context adaptive binary arithmetic coding (CABAC) , which involves three elementary steps: binarization, context modeling, and binary arithmetic coding (Marpe et al. 2003), as the entropy coder. The binarization step maps the non-binary valued syntax elements (SEs), which will be represented in the bitstream and describe how the video sequence can be reconstructed at the decoder, to binary symbols. This step will prolong the encoding pipelines, for it typically maps one element to a bin string. The modeling stage assigns a model probability distribution which was updated using the statistics of the already coded neighboring symbols to binary symbols. In arithmetic coding stage, the actual coding engine is driven by the probability model. Based on recursive interval division and selection, the coding procedure generates a sequence of bits for representing the SEs.

CABAC has the advantage of high coding efficiency. However, these three steps are highly sequential, and induce strong data dependencies (Sze and Budagavi 2012). So, it is difficult to exploit parallelism and pipelining, and makes CABAC a well-known throughput bottleneck in the video codec implementation (Sze and Budagavi 2012; Sole et al. 2012). Typical approaches to fast CABAC rate estimation for mode decision simplify or eliminate the modeling and the coding steps, but leave the binarization step unchanged. The CABAC rate estimator for H.264/AVC introduced in Hahm and Kyung (2010) simplified the context modeling part, and replaced the calculation of the arithmetic coding by a table lookup scheme. It designed multiple lookup tables. Entries in the table were indexed by the probability state indexes which were integers between 0 and 62, and there had a one-to-one correspondence between the entries and a set of predefined representative probability values of the least probable symbol (LPS). By simplifying the modeling and coding steps, the rate estimator yielded about a 30 % reduction in the computational complexity of the R-D evaluation for H.264/AVC (Hahm and Kyung 2010). The fast CABAC rate estimator in Won et al. (2012) also was developed for H.264/AVC, and simplified the coding step by using a lookup table scheme. It designed only one table, which depended on two values, one was an index of the LPS probability, and the other was an indication of whether or not the most probable symbol (MPS) and the current binary to be coded were equal. In Hahm et al. (2009), a rate estimator which approximated the context modeling in CABAC was proposed. It was reported that the estimator reduced about 20 % of the computational complexity of the R-D optimization.

Our objective is to develop a fast CABAC rate estimator for mode decision in HEVC. Based on the assumption of CABAC in HEVC being able to achieve compression close to the entropy of a symbol sequence (Sze and Budagavi 2012), the proposed approach estimates the CABAC rate as a weighted sum of the information generated by the source. All three steps of CABAC, i.e., binarization, context modeling, and binary arithmetic coding, are eliminated. So, the proposed estimator has the advantages of being computationally more efficient and making the encoder better parallelizable.

The remainder of the paper is organized as follows. In “Entropy-based CABAC rate estimation” section, we present an overview of the rate estimation for R-D optimization in HEVC first. Then, the correlation between the entropy and CABAC rate of CUs is evaluated, and the entropy-based CABAC rate estimator is proposed. In “Experimental results” section, some experimental results are demonstrated. Finally, the paper is concluded in “Conclusion” section.

## Entropy-based CABAC rate estimation

### Rate estimation for rate-distortion optimization in HEVC

The HEVC design follows the classic block-based hybrid video coding approach. A picture is partitioned into a sequence of CTUs, which are analogous to macroblocks in previous standards. A CTU may contain only one CU, or may be split into four equal size CUs. In a recursive manner, a CU has a size of $2N \times 2N$ ($N = 8, 16, 32$) can be further split into four smaller units of equal size. The block partition structure of a CTU is quadtree-like. A CU, which is the leaf node of the quadtree, specifies a region sharing the same prediction mode, i.e., intra or inter. The CU consists of a luma coding block (CB) and the corresponding chroma CBs and related syntax elements. Further, a CU can be split into one, two, or four PUs, and a PU defines a region sharing the same prediction information. For intra coded CUs, two possible PU splitting types are supported. For inter coded CUs, eight splitting types are defined. And for skipped CUs, only one PU splitting type (i.e., the same size as the CU) is allowed (Kim et al. 2012). After prediction and compensation, a nested quadtree partitions a CU residual into transform units, each of which defines a region sharing the same transformation.

How to determine the code tree configuration, determine the PU division, and select prediction modes for all CUs in a CTU is a critical problem for improving the coding efficiency. HEVC treats this problem as the so-called rate-distortion optimization (Sullivan and Wiegand 1998), that is,

1 $$\begin{aligned} \min \left\{ D \right\} ,\quad \quad \mathrm{{s.t.}}\quad R \le R_c \end{aligned}$$

where *R* and *D* represent the rate and distortion for a CU, respectively. $R_c$ is the rate constraint. The constrained optimization task in (1) is solved using Lagrangian optimization where the distortion term is weighted against the rate term (Sullivan and Wiegand 1998),

2 $$\begin{aligned} P^*&= \mathop {\arg \min }\limits _{P \in M} \left\{ {J_p } \right\} \nonumber \\ J&= D + \lambda R \end{aligned}$$

where *M* denotes all possible coding parameter set (i.e., the setting of CU size, PU division, and prediction mode, etc.), *J* is the Lagrangian rate-distortion functional, and $\lambda$ is the Lagrangian multiplier, which is usually determined by experiments or by the quantization parameter (QP) (Sullivan and Wiegand 1998). Taking CU depth decision for example, the R-D cost of $CU_i$ (CU in the depth *i*) encoded in the un-split manner will compare with that in the split manner. The problem of coding tree configuration can be implemented by judging whether a CU with each size should be split or not in a recursive way (Xiong et al. 2014).

The rate term *R* in (2) may significantly affect the optimization process. Let *r* denote the ratio between $\lambda R$ and *J* of an encoded CU, i.e., $r = \frac{{\lambda R}}{J}$. Generally, larger *r* value implies that many more bits were coded for representing the SEs for the CU, and higher computational burden had imposed on the CABAC rate estimator. We computed the average *r* value for several test sequences and depicted the results in Table 1. It can be noticed that the average ratio is from 8.2 to 37.5 % (generally, the smaller the CU size, the bigger the ratio value). Before actual entropy coding, the optimization process in (2) should be performed for all candidate CUs, PUs, and TUs to obtain the optimal coding settings for a CTU. So, the computational burden for estimating the rate term is very high, and it is necessary to develop a fast CABAC rate estimator with adequate accuracy for the rate-distortion optimization process.

### The correlation between the entropy and CABAC rate

Suppose there is a source containing a discrete set of independent messages $z_k$ with probabilities $\Pr (k)$. The entropy, which measures the average information generated by all messages in the source, is defined as

3 $$\begin{aligned} H = - \sum \limits _{k = 0}^{L - 1} {\Pr (k) \log _2 \Pr (k) } \end{aligned}$$

where *L* denotes the number of possible different messages. According to Shannon’s noiseless coding theorem (Jain 1988), it is possible to code without distortion a source of entropy *H* bits using an average of $H + \varepsilon$ bits per message, where $\varepsilon$ is an arbitrarily small quantity.

We regard the SEs of a CU as a source. Using the entropy *H* of the SEs used to represent the information of the CU, we can estimate the lower bound on the number of bits required to encode the output of the CU, that is,

4 $$\begin{aligned} R_{\min } = H\sum \limits _k {N\left( {z_k } \right) } \end{aligned}$$

where ${N\left( {z_k } \right) }$ is the occurrence frequency of the elements with value $z_k$.

**Fig. 1:**
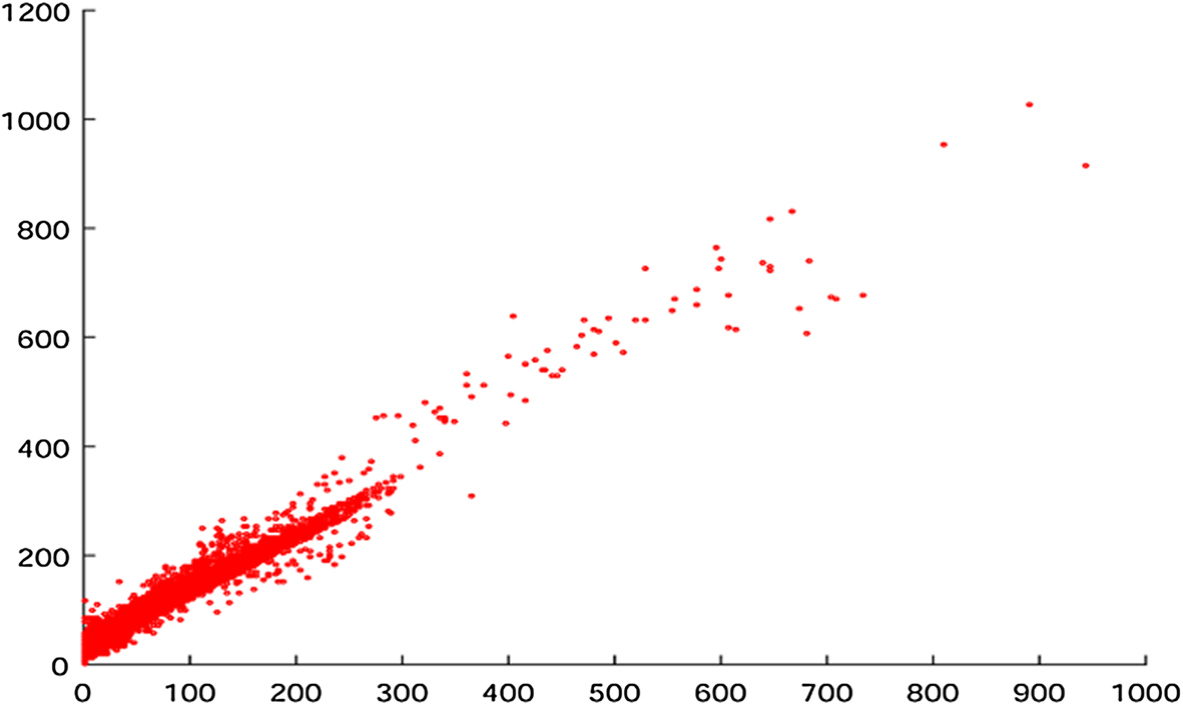
Illustration of the distribution of the sample data $\left\{ {\left( {x_i , y_i } \right) } \right\}$ which were obtained from the first 25 frames of the sequence *BasketballDrill*, where $x_i$ is the estimated lower bound and $y_i$ is the number of actual output bits of the CABAC encoder for a CU

Let $x_i$ and $y_i$ denote the estimated lower bound using (4) and the actual output of the CABAC encoder in HM 13.0 (Kim et al. 2013) for $CU_i$, respectively. We represent them as a paired data $(x_i, y_i)$. The sample set $\{(x_i, y_i)\}$ obtained from the first 25 frames of the test sequence *BasketballDrill* are depicted as points in Fig. 1. We noticed that there might exhibit difference between the estimated lower bound and the actual outputted bits of a CU. However, the experiments also suggested that there was a high correlation between the variables *x* and *y*. For confirming this hypothesis, the correlation between them is quantitatively measured as the correlation coefficient below:

5 $$\begin{aligned} c = \frac{{\sum \nolimits _i {\left( {x_i - \bar{x}} \right) \left( {y_i - \bar{y}} \right) } }}{{\sqrt{\sum \nolimits _i {\left( {x_i - \bar{x}} \right) ^2 } } \sqrt{\sum \nolimits _i {\left( {y_i - \bar{y}} \right) ^2 } } }} \end{aligned}$$

where $\bar{x}$ and $\bar{y}$ are the means of *x* and *y*, respectively. The results for several test sequences are depicted in Table 2.

**Fig. 2:**
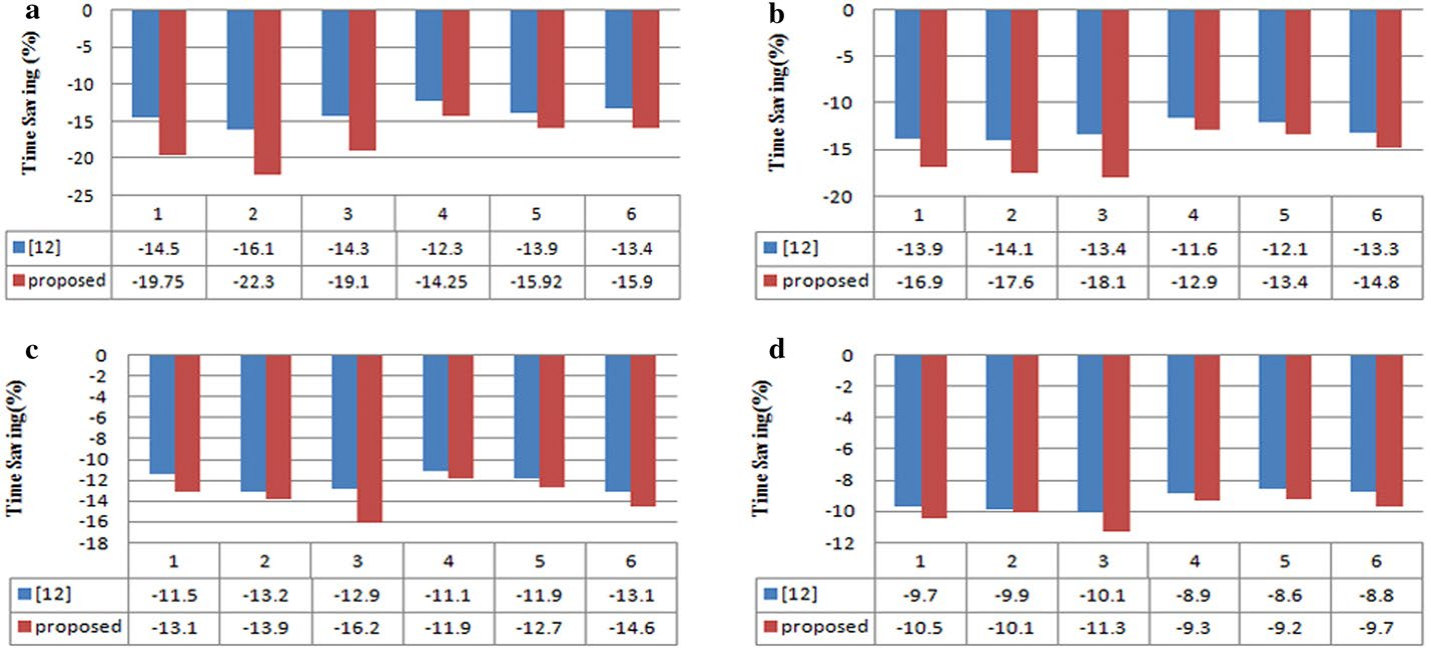
The mean value of the reduction of the time consumption for R-D evaluation (in percent) for sequences in Table 4, **a**–**d** QP = 22, 27, 32, and 37

**Table 1:** Ratio *r* (in percent) for CUs of different sizes for several sequences with QP = 27

Sequence	$32\times 32$	$16 \times 16$	$8 \times 8$
BasketballDrive_1920×1080	10.1	17.2	26.5
BQTerrace_1920×1080	15.1	24.5	36.1
Parkjoy_1920×1080	8.2	24.4	37.5
Vidyo1_1280×720	8.9	18.1	28.4
Video4_1280×720	10.2	18.69	27.9
FourPeople_1280×720	9.2	17.3	29.2

**Table 2:** Illustration of the correlation coefficient for sequences with different QP

Sequence	QP = 22	QP = 37
BasketballDrive_1920×1080	0.9669	0.9711
BQTerrace_1920×1080	0.9764	0.9761
Vidyo1_1280×720	0.9730	0.9769
Video4_1280×720	0.9661	0.9771
FourPeople_1280×720	0.9761	0.9766
KristenAndSara_1280×720	0.9774	0.9774

### The proposed CABAC rate estimator and implementation

In CABAC, binarization maps a given SE to an unique binary string, and different element results different bins with different number of “0” and “1” bits. For the regular coding mode, a particular context model will be chosen, and one of two binary values (0 or 1) will be identified as the MPS, while the other will be identified as the LPS (Sole et al. 2012). Further, a LPS reduces more the interval range, and has a higher probability to generate an output bit than a MPS does. Considering the effect of these two steps, $R_{\min }$ in (4) is not directly adopted as the estimation of the number of coded bits for a CU. Instead, we introduce a vector $\mathbf {w}$ containing the weights corresponding to different SE values to take into account the effect of the binarization and context modeling. The estimator is formulated as the linear regression model below

6 $$\begin{aligned} {\hat{R}} = \mathbf{{w}}^T \mathbf{{u}} = w_0 u_0 + w_1 u_1 + \cdots + w_n u_n \end{aligned}$$

where $\mathbf {w}$ and $\mathbf {u}$ are *L*-dimensional column vectors, and

7 $$\begin{aligned} u_k = \left\{ {\begin{array}{*{20}c} { - N\left( {z_k } \right) \log _2 {h\left( {z_k } \right) } } &{} \quad {h\left( {z_k } \right) \ne 0} \\ 0 &{} \quad {\mathrm{{otherwise}}} \\ \end{array}} \right. \end{aligned}$$

and

8 $$\begin{aligned} h\left( {z_k } \right) = \frac{{N\left( {z_k } \right) }}{{\sum \nolimits _{i = 0}^{L - 1} {N\left( {z_i } \right) } }} \end{aligned}$$

The formulation uses $\mathbf {u}$ as the input regressor vector. It now remains to determine a suitable parameter vector $\mathbf {w}$ that can predict the CABAC rate with acceptable accuracy. We regard the estimator in (6) as a time varying system whose parameters change over time. Since there exists high correlation between the lower bound and the number of actual coded bits of the CABAC encoder, it is reasonable to assume that the variations of the parameters are slow. We address the parameter vector as the state of a linear discrete-time dynamic system.

9 $$\begin{aligned} \mathbf{{w}}_k = \mathbf{{w}}_{k - 1} + e_1 \end{aligned}$$

We embed the parameter updating within the actual CABAC coding process, which is activated to code all the SEs into bitstream after the optimal structure of the CTU and the best mode of the current CU being determined. The relation between the observed measurement and state vector is:

10 $$\begin{aligned} R_k = \mathbf{{w}}_k^T \mathbf{{u}}_k + e_2 \end{aligned}$$

where $R_k$ is the number of actual coded bits for $CU_k$. $e_1$ and $e_2$ represent the process and measurement noise, respectively. They are assumed to be white and with normal probability distributions, that is:

11 $$\begin{aligned}&p\left( {e_1 } \right) \sim N\left( {0,\;\mathbf {Q}} \right) \end{aligned}$$

12 $$\begin{aligned}&p\left( {e_2 } \right) \sim N\left( {0,\;\sigma _e^2 } \right) \end{aligned}$$

The process noise covariance matrix $\mathbf {Q}$ is assumed to be diagonal with very small diagonal entries. This means that state parameters are independent from each other and the variance of a state is small. The variance $\sigma _e^2$ in (12) changes with each measurement, and is defined by

13 $$\begin{aligned} \sigma _e^2 = E\left[ {\left( {R_k - {\hat{R}}_k } \right) ^2 } \right] \end{aligned}$$

The Kalman filter (Catlin 1988), which estimates the state by using a form of feedback control, is employed in our system. The time update (prediction) equations are:

14 $$\begin{aligned} \left\{ {\begin{array}{*{20}l} {{\hat{\mathbf{w}}}}_{k + 1}^{ -} = &{}{{\hat{\mathbf{w}}}}_k \\ {\mathbf{{P}}_{k + 1}^ - = } &{} {\mathbf{{P}}_k + \mathbf {Q}} \\ \end{array}} \right. \end{aligned}$$

where ${{\hat{\mathbf{w}}}}_{k + 1}^ -$ denotes the a priori state estimate, and ${\mathbf{{P}}_{k + 1}^ - }$ is the a priori state error covariance matrix for time update. During the measurement update, the Kalman gain is computed as:

15 $$\begin{aligned} \mathbf{{g}}_{k + 1} = \frac{{\mathbf{{P}}_{k + 1}^ - \mathbf{{u}}_{k + 1} }}{{\mathbf{{u}}_{k + 1}^T \mathbf{{P}}_{k + 1}^ - \mathbf{{u}}_{k + 1} + \sigma _e^2 }} \end{aligned}$$

Then, the a posteriori state estimate is calculated as:

16 $$\begin{aligned} {\hat{\mathbf{w}}}_{k + 1} = {\hat{\mathbf{w}}}_{k + 1}^ - + \mathbf{{g}}_{k + 1} \left( {R_{k + 1} - \mathbf{{u}}_{k + 1}^T {\hat{\mathbf{w}}}_{k + 1}^ - } \right) \end{aligned}$$

And the a posteriori error covariance is:

17 $$\begin{aligned} \mathbf{{P}}_{k + 1} = \left( {\mathbf{{I}} - \mathbf{{g}}_{k + 1} \mathbf{{u}}_{k + 1}^T } \right) \mathbf{{P}}_{k + 1}^ - \end{aligned}$$

where $\mathbf {I}$ denote the identity matrix.

Finally, we would like to make some comments about the proposed CABAC rate estimator here. First, for a CU, the update of the weight vector is only performed once, while the estimator in (6) was evaluated dozens of times for determining the prediction mode, PU partition. Though the update in (14)–(17) are somewhat computationally complicated, it will not degrade the computational efficiency of the estimator more. Second, HEVC has many different SEs. For a SE which was represented as an on/off flag (e.g., merge_flag, cu_split_flag), we simply added one bit to the estimated rate for each flag, while Eq. (6) was applied for other SEs in our experiments.

**Table 3:** Test conditions

Encoder	HM 13.0
Max and min CU sizes	$64 \times 64$ and $8 \times 8$
Max and min TU sizes	$32 \times 32$ and $4 \times 4$
Fast ME	Enabled and the search range is 64
QPs	22, 27, 32, 37

**Table 4:** Test sequences

Class	Sequence no.	Name	Number of coded frames
B ($1920 \times 1080$))	1	BasketballDrive	500
	2	BQTerrace	500
	3	Parkjoy	500
E ($1280 \times 720$)	4	Vidyo1	300
	5	Vidyo4	300
	6	FourPeople	600

## Experimental results

To evaluate the performance of the fast entropy-based CABAC rate estimator, the proposed algorithm was implemented on the HEVC reference software HM 13.0 (Kim et al. 2013) with the HEVC common test conditions (Bossen 2012) which were summarized in Table 3. We encoded three class B sequences with spatial resolution of $1920 \times 1080$ and three class E sequences with resolution of $1280 \times 720$. The test sequences and their sequence number are tabulated in Table 4. Simulations were run on a personal computer with an Intel Core i5-4430 CPU and 4 GB RAM. The operating system was Microsoft Windows 7 64-bit Enterprise edition.

### Time saving

We recorded the time consumption for mode decision for all CUs (that is, the time consumed by *xCompressCU*(), which is the CU analysis module in HM software), and accumulated them. The computational complexity reduction was calculated as follows (Xiong et al. 2014):

18 $$\begin{aligned} \Delta T = \frac{{T_{test} - T_{ref} }}{{T_{ref} }} \times 100\,\% \end{aligned}$$

where $T_{ref}$ denotes the accumulated time consumption of the original HM 13.0 encoder, and $T_{test}$ denotes the time consumed when the fast CABAC rate estimators were adopted for R-D cost evaluation.

For comparison, the algorithm in Won et al. (2012), which was developed for fast rate estimation for H.264/AVC mode decision, was also implemented on HM. The mean value of $\Delta T$ for the tested sequences in Table 4 are summarized in Fig. 2. It shows that the proposed algorithm saves the rate estimation computation from 9.2 to 22.3 %, and 14.5 % on average. The results also indicate that the proposed estimator is computationally more efficient than the previous algorithm. Especially, the performance improvement was higher when the QP was small. The reason for this is that the QP value has an impact on the overall encoding time, and smaller QP implies higher residual energy, and higher computational burden will imposed on the CABAC rate estimator. Under these circumstances, our scheme has the advantage of eliminating all three steps of CABAC coding, while the method in Won et al. (2012) leaves the binarization step unchanged.

### Compression efficiency

We compared the coding performance of the proposed algorithm with the reference software in terms of Bjøntegaard delta rate (BD-Rate), Bjøntegaard delta PSNR (BD-PSNR) (Bjøntegaard 2001). The experimental results were summarized in Table 5. We notice that the proposed estimator slightly increased the BD-rate. For luminance component, the increment is from 1.81 to 2.43 %, and about 2.21 % on average. The increment for chrominance components (Cb and Cr) is from 0.19 to 2.29 %, and 1.19 % on average. However, the complexity reduction and the performance of better parallelizable it provided were worth the expense of the rate increment. From the results, it also can be observed that the BD-PSNR loss is minor. Also, we evaluated the performance of the CABAC rate estimator by the estimation error using the criterion below

19 $$\begin{aligned} \Delta R = \frac{{\left| {R_t - R_r } \right| }}{{R_t + R_r}} \times 100\,\% \end{aligned}$$

where $R_t$ is the estimated rate of our estimator, and $R_r$ is that of the HM software. We calculated the mean value of $\Delta R$ for all CUs, and depict the result in Table 5. The coding performance of the proposed algorithm was also compared with the estimator in Won et al. (2012) in terms of $\Delta T$, BD-Rate and BD-PSNR, and the results were depicted in Table 6.

**Table 5:** Mean value of ${\Delta R}$, ${\Delta T}$, BD-rate increment and BD-PSNR loss compared with the HEVC reference software

Class	Seq. no.	${\Delta R}$ (%)	${\Delta T}$ (%)	BD-Rate (%)			BD-PSNR (dB)		
				Y	Cb	Cr	Y	Cb	Cr
B	1	7.67	15.07	2.43	1.79	1.85	−0.09	−0.03	−0.06
	2	5.11	15.98	2.28	1.63	0.70	−0.08	−0.03	−0.02
	3	6.62	16.17	2.36	2.29	2.15	−0.11	−0.09	−0.07
E	4	4.14	12.09	2.18	0.81	1.01	−0.11	−0.03	−0.03
	5	5.88	12.81	1.81	0.89	1.15	−0.09	−0.03	−0.05
	6	3.91	13.75	2.14	1.22	0.19	−0.12	−0.04	−0.01

**Table 6:** Coding performance (mean value of $\Delta T$, BD-Rate and BD-PSNR) of the proposed method compare with the method in Won et al. (2012)

Class	Seq. no.	${\Delta T}$ (%)	BD-Rate (%)			BD-PSNR (dB)		
			Y	Cb	Cr	Y	Cb	Cr
B	1	2.83	0.43	0.31	0.28	−0.03	0.01	−0.02
	2	2.07	0.38	0.20	−0.08	−0.02	−0.00	0.01
	3	2.91	0.36	0.21	0.19	−0.02	−0.00	0.01
E	4	1.03	0.28	−0.09	0.09	−0.01	−0.01	−0.01
	5	1.06	0.31	0.06	0.15	−0.02	0.01	−0.02
	6	1.43	0.14	−0.11	−0.07	−0.22	0.01	0.01

## Conclusion

A fast entropy-based CABAC rate estimation algorithm, which is applicable to rate-distortion optimization in HEVC, is proposed in this paper. The syntax elements of a CU are regarded as a source containing a discrete set of independent messages. The implicit relation between the entropy of the source and the number of coded bits (i.e., the output of the CABAC encoder) for the CU is investigated. Exploiting the correlation between these two values, the proposed approach estimates the CABAC rate as a weighted sum of the information generated by the source. The weight vector, which is employed to compensate the effect of the binarization and context modeling steps, is addressed as the state of a linear discrete-time dynamic system. The weights are adaptively updated within the actual CABAC encoding process, which is activated to encode all the SEs into bitstream after the best mode of the current CU being determined, using the Kalman filtering.
